# An Aromatic Aldehyde Synthase Controls the Synthesis of Hydroxytyrosol Derivatives Present in Virgin Olive Oil

**DOI:** 10.3390/antiox8090352

**Published:** 2019-09-01

**Authors:** Rosario Sánchez, Lourdes García-Vico, Carlos Sanz, Ana G. Pérez

**Affiliations:** Department of Biochemistry and Molecular Biology of Plant Products, Instituto de la Grasa (CSIC), 41013 Sevilla, Spain

**Keywords:** *Olea europaea*, hydroxytyrosol, aromatic aldehyde synthase, olive fruit, phenolic compounds, quality, virgin olive oil

## Abstract

The phenolic composition of virgin olive oil (VOO) is strongly determined by the content and distribution of secoiridoid phenolic glucosides present in the olive fruit. Among them, oleuropein is the most abundant in olive mesocarp and is characterized by containing an hydroxytyrosol residue in its chemical structure. Hydroxytyrosol-containing molecules are those that exhibit the most important biological activities in virgin olive oil. In this regard, we identified an aromatic aldehyde synthase gene (*OeAAS*) from an olive transcriptome, which was synthesized, expressed in *Eschrichia coli*, and purified its encoded protein. The recombinant OeAAS is a bifunctional enzyme catalyzing decarboxylation and amine-oxidation reactions in a single step. OeAAS displays strict substrate specificity for l-DOPA to form 2,4-dihydroxyphenylacetaldehyde, the immediate precursor of hydroxytyrosol. In addition to the biochemical characterization of the enzyme, the expression analysis carried out in different olive cultivars and ripening stages indicate that *OeAAS* gene is temporally regulated in a cultivar-dependent manner. High correlation coefficients were found between *OeAAS* expression levels and the phenolic content of olive fruits and oils, which supports a key role for *OeAAS* in the accumulation of hydroxytyrosol-derived secoiridoid compounds in olive fruit and virgin olive oil.

## 1. Introduction

The phenolic components of virgin olive oil (VOO) are directly associated to the organoleptic [1,2] and functional properties [3,4] of this highly appreciated Mediterranean food. The content of phenolic glycosides initially present in the olive fruit tissues, transformed by hydrolytic and oxidative enzymes (β-glucosidase, polyphenol oxidase, and peroxidase) during the oil extraction process, strongly affects the biosynthesis and accumulation of phenolic compounds in VOO [5,6,7].

The major phenolic glycosides found in olive (*Olea europaea* L.) are oleuropein and ligstroside. These compounds have a rather complex secoiridoid structure, containing both monoterpenoid and phenolic residues. Oleuropein, which is the most abundant secoiridoid glycoside in olive mesocarp, is the ester of elenolic acid glycoside and hydroxytyrosol (3,4-DHPEA), an alcohol of phenylpropanoid origin. Ligstroside has a very similar structure but is esterified with the monohydroxy phenylalcohol tyrosol (*p*-HPEA). The secoiridoid derivatives resulting from the enzymatic hydrolysis of these olive glycosides, occurring during the oil extraction process, are the dialdehydic forms of decarboxymethyloleuropein and ligstroside aglycones (3,4-DHPEA-EDA and *p*-HPEA-EDA, respectively) and the aldehydic forms of oleuropein and ligstroside aglycones (3,4-DHPEA-EA and *p*-HPEA-EA, respectively). These secoiridoid derivatives are the most abundant phenolic components found in VOO [8]. Molecules containing hydroxytyrosol are those that exhibit the most important biological activities and highest effectivity as chronic disease preventive agents, due to their capacity to reduce chronic inflammation and oxidative damage and their antiproliferative properties [9,10,11]. These scientific evidences led the European Union to approve a health claim on olive oil polyphenols, which may be applied to oils containing at least 250 ppm of hydroxytyrosol and its derivatives [12].

**Figure 1:**
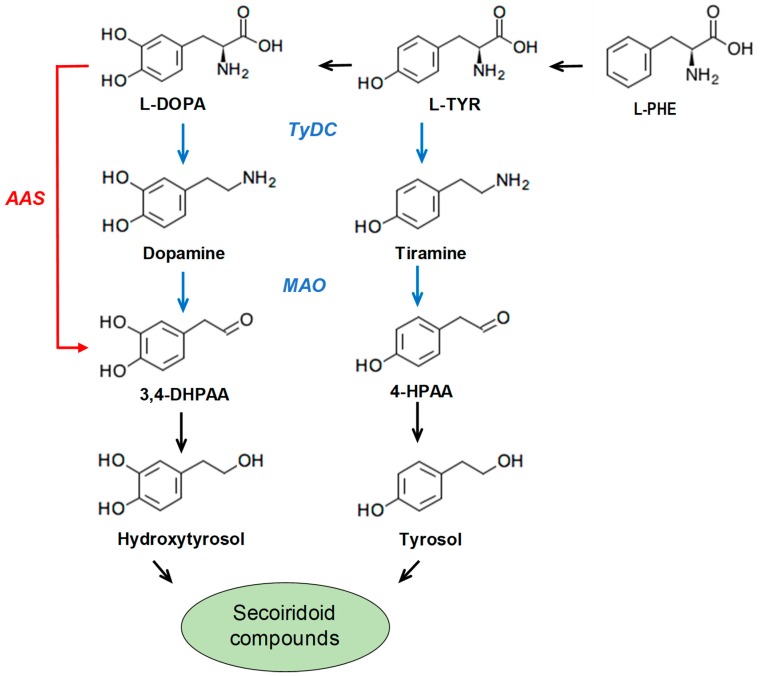
Proposed biosynthesis pathway for the phenolic alcohols hydroxytyrosol and tyrosol, which constitute the phenol moiety of secoiridoid compounds in olive. Abbreviated intermediate metabolites and enzymes are: 3,4 dihidroxyphenylacetaldehyde (3,4-DHPAA); 4-hydroxyphenylacetaldehyde (4-HPAA); Tirosine/Dopa decarboxylase (TyDC); Monoamino oxidase (MAO); Aromatic aldehyde synthase (AAS).

Despite the growing interest in secoiridoid derivatives as functional components of VOO or new functional ingredients, the natural biosynthesis and degradation pathways of these compounds in olive are not yet fully understood [13]. Some biotechnological methods have been reported for the conversion of tyrosol into hydroxytyrosol [14,15], and more recently, whole cell catalytic methods for the synthesis of tyrosol and hydroxytyrosol in engineered *Escherichia coli* have been reported [16,17]. However, the specific genes controlling the biosynthesis pathway for hydroxytyrosol and tyrosol in olive have not been fully elucidated yet. The proposed biosynthesis pathway for the phenolic alcohols hydroxytyrosol and tyrosol is shown in Figure 1.

In the biosynthetic pathway postulated by Lan et al. [18] in *Rhodiola crenulata*, tyrosol is generated from tyrosine through sequential decarboxylation, oxidative deamination, and aldehyde reduction reactions, catalyzed by three discrete enzymes: Tyrosinedecarboxylase (TyDC; EC 4.1.1.25), monoamine oxidase (MAO; EC 1.4.3.4), and 4-hydroxyphenylacetaldehyde reductase (4HPAR; aryl-alchol dehydrogenase EC 1.1.1.90), respectively. Unlike the previously proposed pathway involving separate decarboxylation and deamination enzymatic steps from tyrosine to the key intermediate 4-hydroxyphenylacetaldehyde (4-HPAA), Torrens-Spence et al. [19] recently reported that medicinal plants of the *Rhodiola* genus, which display high levels of tyrosol 8-O-glucoside, contain a pyridoxal phosphate (PLP)-dependent aromatic aldehyde synthase (AAS) that directly converts tyrosine into 4-HPAA (Figure 1). TyDCs, along with tryptophan decarboxylases (TDC; EC 4.1.1.27) and AASs, encompass a large family of PLP-dependent enzymes generally known as the aromatic amino acid decarboxylase (AAAD) family [20,21]. This family shows a great divergence in terms of activity but a high amino acid sequence identity [22]. However, two studies have recently characterized the specific primary sequence features that correspond to TyDC, TDC, and AAS [23,24]. Thus, a glycine to serine substitution at position 370 is associated to preference for indolic/phenolic amino acids as substrates and a tyrosine/phenylalanine substitution at position 350 seems to be related to pure decarboxylation versus aldehyde synthase activities [23].

In the framework of the characterization of the genes and enzymes responsible for the biosynthesis of hydroxytyrosol derivatives in olive fruit, the purpose of the present work is the study of the key metabolic step in this biosynthesis, which uses DOPA as substrate to form 2,4-DHPAA, the precursor of hydroxytyrosol. The expression levels of the *AAS* gene in the olive fruit, together with the catalytic properties of the encoded protein, are consistent with its participation in the biosynthesis of hydroxytyrosol, whose derivatives are the most important class of phenolic compounds present in VOO.

## 2. Materials and Methods

### 2.1. Plant Material

Seven olive cultivars from the Core-36 olive collection established by Belaj et al. [25] and maintained at the World Olive Germplasm Collection (WOGC, IFAPA Alameda del Obispo, Cordoba, Spain), having extremely different phenolic profiles [8], were used in this study: Picual, Menya, Shengeh, Piñonera, Klon, Dokkar, and Abou kanani.

Olive trees from these cultivars were grown in the same edapho-climatic conditions, using drip irrigation and standard cultural practices, at the experimental orchards of IFAPA Alameda del Obispo (Córdoba, Spain). The fruits were harvested at different stages of ripening, which were identified both by the color of the fruit and by the weeks after flowering (WAF) as follows: Stage I, green (17–19 WAF); stage II, yellow-green (23 WAF); stage III, turning (28–30 WAF); and stage IV, black fully ripe (35 WAF). Olive tissues to be used for RNA extraction were frozen in liquid nitrogen immediately after harvesting and stored at −80 °C.

### 2.2. RNA Extraction, cDNA Library Construction, and Sequencing

Total RNA was extracted from 1–2 g of frozen olive mesocarp tissues, from fruits harvested from two different olive trees of cultivars Picual, Menya, Shengeh, Piñonera, Klon, Dokkar, and Abou kanani, and two different ripening stages (II and IV), using the Spectrum Plant Total RNA Kit (Sigma-Aldrich, St. Louis, MO, USA) according to the supplier’s instructions. Three biological replications per sample were used. RNA concentration and purity were tested using nanodrop-photometric quantification (Thermo Scientific, Waltham, MA, USA). RNA samples were sent to Centro Nacional de Análisis Genómico (CNAG, Barcelona, Spain) for library preparation and paired end sequencing (2 × 75 bp) on Illumina Hi-Seq 2000 platform (Illumina Inc., San Diego, CA, USA) to generate the raw data (average 48 million read-pairs/sample) of the samples. The high-quality reads for the samples were then assembled into transcripts using RNA-Seq assembler Trinity [26]. The assembled sequences were annotated against the olive genome data base OE6.OLIVEFAT (CNAG, Barcelona, Spain) and with the help of Blast2GO program [27].

### 2.3. Isolation of An AAS Full-Length cDNA Clone

Blast2GO suite [27] was used to find homologous sequence candidates to the aromatic acetaldehyde synthase (AAS) from *Rhodiola rosea* (AUI41112.1) and *Petroselinum crispum* (Q06086.1) against the olive transcriptome generated. The only coding sequence found (*OeAAS*, OE6A000744) was synthesized *de novo* according to the codon usage of *Escherichia coli* (GenScript, Piscataway, NJ) and cloned into pET-28a(+)-TEV vector as *Nde*I-*Xho*I fragment (OeAAS construct).

### 2.4. Gene Expression Analysis by RT-QPCR

Gene expression analysis was performed by real-time quantitative PCR (RT-QPCR). For this purpose, total RNA extraction from olive mesocarp tissues was carried out as described above, and the corresponding cDNA libraries were synthesized using the Ready-To-Go T-Primed First Strand Kit (Amersham Bioscience, Roosendaal, The Netherlands). Specific pair of primers for gene amplification of the olive *OeAAS* gene were designed (Table S1) and the cDNAs were subjected to RT-QPCR using SYBR Green I (SsoAdvanced^TM^ Universal SYBR^®^ Green Supermix, BioRad, Hercules, CA, USA) in a CFX96 Touch System (BioRad) to monitor the resulting fluorescence. The reaction mixture was heated to 95 °C for 30 s before subjecting it to 40 PCR cycles consisting of: 95 °C for 15 s; 56 °C for 15 s; and 72 °C for 15 s. Efficiency curves were drawn up for each pair of primers using sequential dilutions of cDNA. The Pfaffl method [28] was applied using the BioRad CFX Maestro 1.0 Software to calculate comparative expression levels between samples. Three olive genes were selected as reference genes according to previous studies [29,30] and were validated in our samples with the GeNorm software included in the BioRad CFX Maestro 1.0 software: Elongation factor-1-alpha (*OeEF1α*), glyceraldehyde-3-phosphate dehydrogenase (*OeGAPDH*), and serine/threonine protein phosphatase 2A (*OePP2A*) (olive genome data base annotation OE6.OLIVEFAT accession numbers OE6A045598, OE6A105640, and OE6A097517, respectively). Three biological and two technical replicates were obtained from each sample. Statistical significance was set at a level of *p* < 0.05 (Tukey’s test).

### 2.5. Heterologous Expression of OeAAS in Escherichia coli and Purification of OeAAS Protein

The *E. coli* strain BL21 (DE3) lacIq containing the *OeAAS* construct was grown at 37 °C in Luria Bertani media (LB), NaCl 0.5 M, to OD_600_ of 0.6, induced with 0.4 mM isopropyl-b-d-thiogalactoside (IPTG), and allowed to grow for an additional 20 h at 18 °C. Cells were harvested by centrifugation, washed with PBS buffer (137 mM NaCl, 2.7 mM KCl, 10 mM Na_2_HPO4, and 1.8 mM KH_2_PO4), resuspended in lysis buffer (50 mM Tris, pH 8.0, 0.5 M NaCl, 40 mM imidazole, 0.5 mM DTT, 1mM PMSF, and 200 µM PLP), and lysed by sonication. The resulting crude protein lysate was clarified by centrifugation prior to nickel–nitrilotriaetic acid (Ni–NTA) purification (His GraviTrap affinity columns, GE Healthcare, Chicago, IL, USA). After loading the clarified lysate, His-tagged recombinant protein-bound Ni–NTA resin was washed with 20 column volumes of wash buffer (50 mM Tris, pH 8.0, 0.5 M NaCl, 60 mM imidazole, and 0.5 mM DTT) and eluted with 3 column volume of elution buffer (50 mM Tris, pH 8.0, 0.5 M NaCl, 250 mM imidazole, and 0.5 mM DTT). Imidazole was removed on a PD-10 (Sephadex G-25, GE Healthcare, Buckinghamshire, UK) desalting column. Desalted protein solution was concentrated using a Vivaspin centrifugal concentrator (MWCO 50 kDa, Merck, Darmstadt, Germany) and stored at 4 °C in storage buffer (20 mM Tris, pH 8.0, 25 mM NaCl, 0.5 mM DTT, and 200 µM PLP). This purified and concentrated preparation was used for OeAAS catalytic characterization. Purity of the recombinant protein was evaluated by SDS-PAGE and densitometry analysis (ChemiDoc Imaging System, BioRad) as previously described [31]. Concentration of the purified recombinant protein was determined by Bio-Rad protein assay kit using bovine serum albumin as a standard.

### 2.6. OeAAS Activity Assay

The AAS enzyme assays were performed in 100 μL of reaction buffer (50 mM sodium phosphate, pH 6.8, 200 μM PLP) containing 7 μg of recombinant OeAAS. Reactions were carried out with a range of amino acid substrate concentrations (0.05–5 mM) at 30 °C for 20 min prior quenching with 100 μL of 0.8 M formic acid. The reaction mixture was centrifuged and the supernatant was analyzed by high-performance liquid chromatography (HPLC). Analysis of substrates and reaction products was performed in a Beckman Coulter HPLC system equipped with a DAD System Gold 168 detector and a Superspher RP 18 column (4.6 × 250mm, particle size 4 μm: Dr. Maisch GmbH, Ammerbuch, Germany). The amount of product in reaction mixtures containing different concentrations of substrates were quantitated based on a standard curve generated using authentic 2,4-DHPAA, 4-HPAA, or phenyl acetaldehyde (PAA) standards. Identification of compounds was confirmed by HPLC/ESI-qTOF-HRMS on a liquid chromatograph Dionex Ultimate 3000 RS U-HPLC liquid chromatograph system (Thermo Fisher Scientific, Waltham, MA, USA) equipped with a similar column and elution program.

### 2.7. Sequence Alignment and Phylogenetic Analysis

The multiple sequence alignments of plant AAS amino acid sequences were calculated using the ClustalX program and displayed with GeneDoc. Phylogenetic tree analysis was performed using the neighbor-joining method implemented in the Phylip package using Kimura’s correction for multiple substitutions and a 1000 bootstrap data set. MEGA5 was used to display the tree. The conserved domains in the deduced amino acid sequences were analyzed using the NCBI Conserved Domain Search (https://www.ncbi.nlm.nih.gov/cdd/) and Pfam software (http://pfam.xfam.org/search/sequence). Accession numbers of the different AAS and TyDC included in the analysis are listed as supplementary data.

### 2.8. Olive Oil Extraction

Olive oil extraction from olive fruits (2–3 kg) was performed by means of an Abencor extractor system (Comercial Abengoa, S.A., Seville, Spain) as previously described [32]. This system comprises a stainless-steel hammer mill operating at 3000 rpm equipped with a 5 mm sieve, a kneader operated for 30 min at 28 °C, and a basket centrifuge for the olive paste running at 3500 rpm for 1 min. The oils were decanted, paper-filtered, and stored under nitrogen atmosphere at −20 °C.

### 2.9. Extraction and Analysis of Fruit and VOO Phenolic Compounds

Fruit phenolic compounds were extracted according to a previously developed protocol [5] using dimethyl sulfoxide (6 mL/g of fruit) with syringic acid (24 mg/mL) as internal standard. The extracts were filtered through a 0.45 μm nylon and kept at −20 °C until HPLC analysis. VOO phenolics were isolated by solid phase extraction (SPE) on a diol-bonded phase cartridge (Supelco, Bellefonte, PA, USA) following a previously described procedure [5]. An aliquot (0.5 mL) of a methanol solution containing *p*-hydroxyphenyl-acetic acid and *o*-coumaric acid was added to each oil sample (2.5 g) as internal standards before the extraction.

Phenolic extracts from both fruits and oils were analyzed on a Beckman Coulter HPLC system equipped with a System Gold 168 DAD detector following a previously described methodology [32]. A Superspher RP 18 column (4.6 × 250 mm, particle size 4 μm; Dr. Maisch GmbH, Germany) at flow rate 1mL/min and a temperature of 35 °C was used for all the analyses. For the analysis of VOO phenolics, the quantification of verbascoside, flavones, and ferulic acid was done at 335 nm, while the rest of phenolic components were quantitated at 280 nm. Response factors were calculated for each phenolic compound. Identification of compounds was confirmed by HPLC/ESI-qTOF-HRMS on a liquid chromatograph Dionex Ultimate 3000 RS U-HPLC liquid chromatograph system (Thermo Fisher Scientific, Waltham, MA, USA) equipped with a similar column and elution program [33]. Mass spectra were acquired in MS fullscan mode and data were processed using TargetAnalysis 1.2 software (Bruker Daltonics, Bremen, Germany).

### 2.10. Extraction and Analysis of Free Amino Acids in Olive Fruit

Olive mesocarp tissues were ground and freeze-dried to remove moisture. Subsequently, acetone powders were obtained from lyophilized tissue according to Romero-Segura et al. [31]. Free amino acids were extracted from acetone powders and derivatized with diethyl ethoxymethylenemalonate and analyzed by HPLC (using dl-α-aminobutyric acid as an internal standard) according to Alaiz et al. [34].

## 3. Results and Discussion

### 3.1. Identification and Molecular Characterization of An AAS Gene in Olive

A blast search using AAS sequences from *Petroselinum crispum* and *Rhodiola rosea* [19,35] as the query against the olive transcriptome generated *de novo* for this study allowed the identification of a number of transcripts that show a high degree of similarity to previously described aromatic amino acid decarboxylases from plants. Four of these transcripts, having the highest homology, appropriate length, and low e-values, were studied, looking for the sequence motifs determinants of the different catalytic mechanisms displayed by the enzymes of the AAAD family [23,24]. The alignment of their deduced amino acid sequences (Figure S1) showed that only one of the transcripts included the phenylalanine residue firstly identified at position 350 in *Papaver Somniferum* and associated to enzymes with AAS activity, which produce both decarboxylation and amine-oxidation of the aromatic amino acids [23]. Meanwhile, the other three, having a tyrosine residue at the same position, likely catalyze only decarboxylation reactions and were identified as TyDC. One of these putative TyDCs (olive genome data base annotation OE6.OLIVEFAT accession number OE6A082511) shares 100% homology with a TyDC (GeneBank accession number AFS28699) previously identified by Alagna et al. [36]. The transcript putatively encoding for an AAS enzyme is a full-length cDNA of 2280 bp containing a coding region of 1464 bp that was named *OeAAS* (GeneBank Accession number MN216342). The product of this putative *AAS* gene is a protein with 487 amino acid residues, a calculated molecular mass of 53.9 kDa and a p*I* of 6.03. The predicted protein showed conserved sequence motifs characteristic of other plant AAADs and group II PLP-dependent amino acid decarboxylases, including the PLP-binding lysine residue (Figure S1). Analysis of the OeAAS deduced protein sequence with target prediction tools WolfPSORT and TargetP did not show signal peptides at its N *termini*, which suggests a cytosolic localization for the protein, similar to what has been reported for other AAS proteins [21].

A phylogenetic analysis was carried out with diverse sequences from the AAAD family, including the few AAS identified so far in plants (Figure S2). Surprisingly, OeAAS shares greater amino acid identity with the protein from *Capsicum annuum* (83%) initially described as TyDC, than with other previously characterized AAS proteins, such as those from *Arabidopsis thaliana* (76%), *Rhodiola rosea* (74%), or *Petunia hybrida* (56%). Besides, the identity with a previously reported olive protein tentatively identified as TyDC (AFS28699.1) was also low (59.7%). In this sense, it is possible that some plant TyDCs had been functionally mischaracterized due to the method used for the activity assay, usually detection of CO_2_ release, which cannot distinguish AAS activity from TyDC activity [23].

### 3.2. Purification and Biochemical Characterization of OeAAS

**Figure 2:**
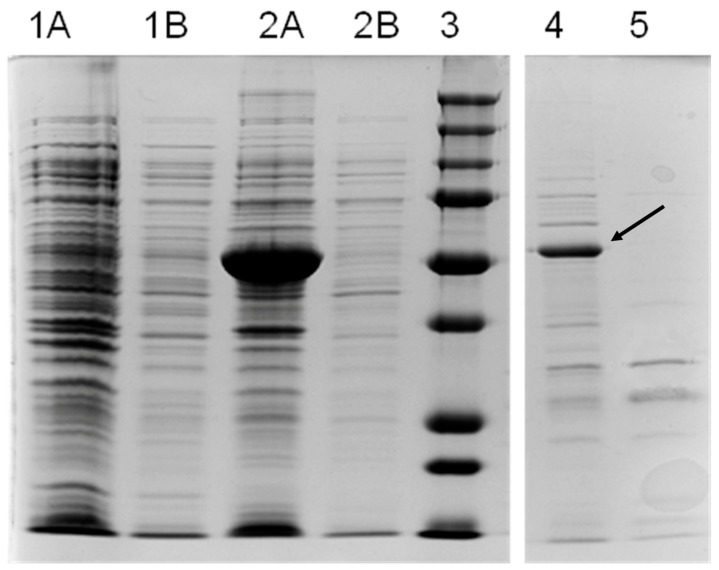
SDS-PAGE of crude extracts and purified preparation of *E. coli* expressing the *Oe*AAS gene. Lanes 1: Crude extract of *E coli,* with empty pET-28a (+) vector as control, pellet fraction (**1A**), and soluble fraction (**1B**); Lanes 2: Crude extract of transformed *E. coli* with the *OeAAS* construct, pellet fraction (**2A**), and soluble fraction (**2B**); Lane 3: Molecular mass markers; Lane 4: Recombinant OeAAS purified preparation (7 µg total protein). The arrow indicates the recombinant protein with a calculated MW of 54 kDa; Lane 5: Purified protein preparation from control *E. coli* (7 µg).

To verify the functional identity of the *OeAAS* gene, we cloned the full-length open reading frame corresponding to the olive *AAS* putative gene into *E. coli* and expressed it as described in the Material and Methods section. Despite the different expression temperatures and different IPTG concentrations tested, most of the recombinant protein was found in the bacterial pellet fraction as inclusion bodies. However, after optimization of the induction process and simple purification and concentration steps, it was possible to obtain sufficient soluble protein for the catalytic characterization of the AAS enzyme (Figure 2). The molecular weight of the recombinant OeAAS calculated by gel electrophoresis was 55 KDa. This value, coincident with the theoretical molecular weight predicted from the amino acid sequence, is quite similar to those reported for other similar enzymes, such as those of arabidopsis or petunia, although both native enzymes have different structures, dimeric and tetrameric, respectively [21].

OeAAS seems to be a PLP-dependent enzyme, since the absence of PLP during purification or in the reaction medium resulted in a total loss of enzyme activity. The addition of PLP in the extraction buffer and at the end of the purification and concentration processes restored the activity. This is also a feature common to all previously reported plant AASs [19,21,23,35].

**Figure 3:**
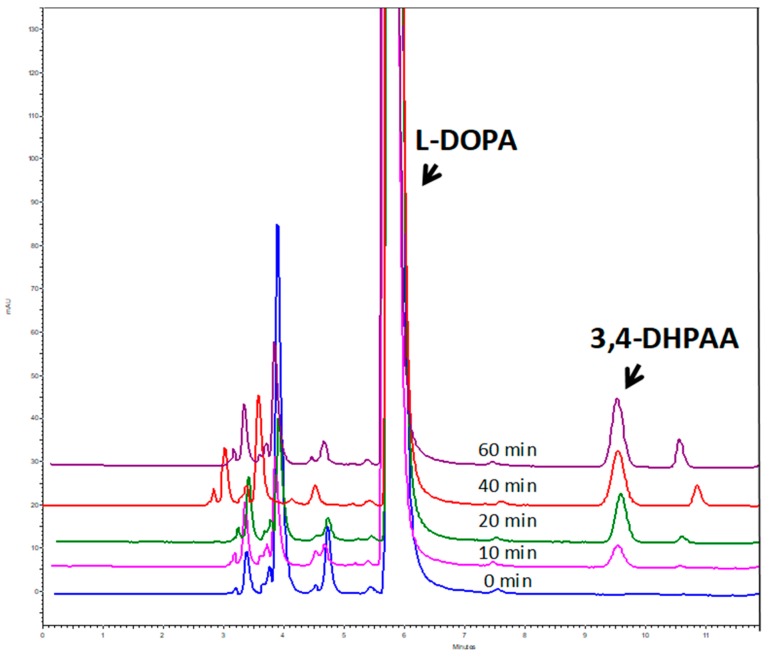
Time course of the formation of 2,4-DHPAA after incubation of recombinant OeAAS with l-DOPA. AAS activity was assayed as indicated in Material and Methods at different incubation times.

The purified recombinant OeAAS produced was evaluated for its ability to catalyze the PLP-dependent decarboxylation/amine-oxidation using the aromatic amino acids l-Phe, l-Tyr and l-DOPA as substrates, through detection and quantification of their reaction products (PAA, 4-HPAA, and 3,4-DHPAA respectively) by HPLC-DAD/MS analysis according to what is described in Material and Methods section. Results showed that the enzyme displayed strict substrate specificity for l-DOPA, forming 3,4-DHPAA as a unique reaction product, with no dopamine detected in the reaction medium (Figure 3). These findings are consistent with the oxidative decarboxylation mechanism proposed for *Petunia* AAS by Kaminaga et al. [21], who could not detect the amine product either. No aromatic aldehydes (PAA, 4-HPAA) or the corresponding intermediate amine products were formed when OeAAS was incubated with l-Phe and l-Tyr as substrates. Among the AASs previously described, only the enzyme from *Petunia hybrida* seems to have such a high specificity, only being active in this case with l-Phe [21].

Optimal pH for recombinant OeAAS enzyme activity using l-DOPA as substrate was measured at 30 °C in a range of pH (4–10). OeAAS displayed a broad pH spectrum of enzymatic activity, showing the highest activity at pH 6.8.

Kinetic characterization of the purified recombinant OeAAS reveled an apparent *K*_m_ for l-DOPA of 0.15 mM and a *V*_max_ of 98 nmol/min.mg. This *K*_m_ value is lower than those previously reported for other plant AAS proteins, indicating a higher affinity for l-DOPA. Thus, *Arabidopsis* AAS (AtAAS) is active with l-Phe and l-DOPA, but not with l-Tyr, having apparent *K*_m_ values of 4.12 and 0.55 mM, respectively, and shows a rather broad optimum pH from 7 to 8.5 [37]. The enzyme from parsley (*Petroselinum crispum*) also exhibited higher *K*_m_ values for l-DOPA (1.40 mM) and for l-Tyr (0.46 mM). The latter seems to be its physiological substrate [35].

To determine the availability of physiological substrates within the olive fruit, the profile of free amino acids was investigated in the olive cultivars selected for this study. Amino acid analysis revealed that l-DOPA was the most abundant aromatic amino acid in most olive cultivars analyzed in this study (data not shown). Thus, the mean concentration of l-DOPA along the ripening of Picual, Menya, Shengeh and Piñonera cultivars, is 194.1 µg/g dry weight (DW), which is 15 times higher than the mean contents of l-Tyr and l-Phe (12.2 and 13.0 µg/g DW, respectively). The highest content of aromatic amino acids along the ripening of these selected olive cultivars was usually found in stage III fruits. At this ripening stage, the highest content of l-DOPA was found in cultivar Picual, with 436 µg/g DW and significantly lower levels of l-Tyr (20 µg/g DW) and l-Phe (26 µg/g DW). These data, together with the strict specificity of OeAAS for l-DOPA, suggest that the synthesis of tyrosol and hydroxytyrosol may not share a common pathway in olive and that, therefore, OeAAS can play a key role in the production of secoiridoid derivatives of hydroxytyrosol, which are quantitatively the main phenolic compounds in olive fruit and in virgin olive oil. A similar conclusion was raised for parsley AAS in relation to its specificity toward l-Tyr and the abundance of this amino acid in parsley [35].

### 3.3. Developmental Expression of Olive AAS Gene Is Cultivar Dependent

**Figure 4:**
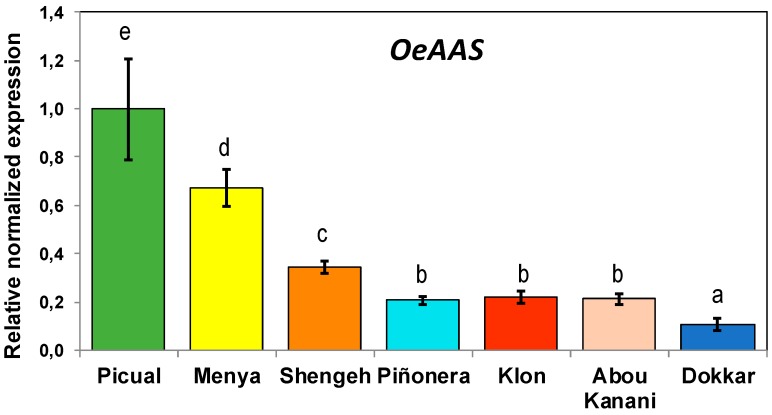
Relative expression levels of olive AAS gene in the mesocarp tissue of Picual, Menya, Shengeh, Piñonera, Klon, Abou kanani, and Dokkar fruits harvested at the commercial maturity stage for olive oil extraction (stage III). Data are mean ± SD. Three biological and two technical replicates were obtained for each sample. Different letters indicate statistically significant differences according to Tukey’s test (*p* ≤ 0.05).

**Figure 5:**
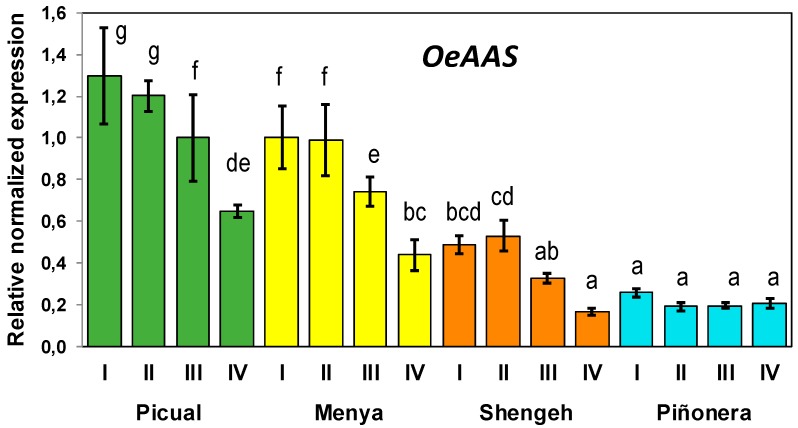
Relative expression levels of olive AAS gene in the mesocarp tissue of olive fruits (Picual, Menya, Shengeh, Piñonera cultivars) along fruit ripening. Data are mean ± SD. Three biological and two technical replicates were obtained for each sample. Different letters indicate statistically significant differences according to Tukey’s test (*p* ≤ 0.05).

The expression levels of *OeAAS* were measured by RT-QPCR, using specific primers and the reference genes described in the Material and Methods section, in fruits from seven selected olive cultivars featuring differences in phenolics contents (Figure 4). Fruits were harvested at the commercial maturity stage usually used for VOO extraction (stage III). Maximum expression levels were found in cultivar Picual, followed by Menya and Shengeh, with significantly lower levels in Piñonera, Klon, Abou kanani, and Dokkar cultivars. In addition, it was found that *OeAAS* expression is temporarily regulated (Figure 5). Expression levels were measured in the fruits from cultivars Picual, Menya, Shengeh, and Piñonera at different fruit ripening stages. Maximum transcription was observed in green and yellow-green fruits (stage I and stage II; 17–19 and 23 WAF, respectively). In most cultivars, the *OeAAS* expression levels showed a clear decrease along fruit development and ripening, with the exception of Piñonera, in which nonsignificant variations in the *OeAAS* expression levels were detected along fruit development and ripening. Related to the olive oil phenolic biochemistry, in a previous study, we found a cultivar-dependent expression and higher expression levels in green fruits for the olive β-glucosidase gene involved in the hydrolysis of oleuropein [38]. However, in this case, a second peak of gene expression was found in some cultivars once the olive fruit ripening had started.

### 3.4. Correlation of OeAAS Expression with Phenolic Accumulation

Once it was seen that the *OeAAS* gene seems to play a key role in the specific synthesis of hydroxytyrosol derivatives in olive fruit and that it is cultivar and temporarily regulated, we studied the relationship that the level of its expression had with the accumulation of secoiridoid phenolic compounds in the olive fruit and in their corresponding VOOs. As shown in Table 1, the total phenolic content of the olive fruit showed a pattern similar to that of the *OeAAS* gene expression, decreasing markedly as the process of fruit ripening progresses. This pattern has been already described for several olive cultivars [39,40]. Only two specific phenolic glucosides showed and opposite trend: Hydroxytyrosol-glucoside, whose increase could be explained by degradation of oleuropein, [40] and demethyloleuropein, which increases in cultivars Piñonera and Menya, probably due to the conversion of oleuropein into demethyloleuropein, as suggested by Gómez-Rico et al. [39]. In most cultivars, the decrease in oleuropein, the most relevant phenolic in the Oleaceae family, parallels the decrease in total phenolics. However, there are significant differences among cultivars. Thus, cultivars Menya and Picual displayed the highest phenolic contents with around 37,000 µg/g in green fruits (stage I) and around 35,000 µg/g in yellow-green fruits (stage II). These olive cultivars also showed the highest expression levels for *OeAAS* (Figure 5). The phenolic content of Piñonera fruits could be described as medium-low, although a quite high phenolic content was found at stage I. On the contrary, the phenolic content of Shengeh fruits could be described as low at any ripening stage. The phenolic composition of the VOOs obtained from fruits harvested during ripening (stages II, III, and IV; oil extraction was not possible from fruits at the green stage) were also noticeably different among cultivars (Table 2).

In general, those cultivars having fruits with high phenolic content gave rise to oils with high phenolic content. Thus, the oils having maximum phenolic contents are those from Picual and Menya. This is in agreement with results found in a previous study with a subset of olive cultivars from the World Olive Germplasm Collection (IFAPA Alameda del Obispo, Cordoba, Spain), in which a very good correlation coefficient (*r* = 0.82) was found for fruit phenolic content and total VOO phenolic content [8]. Similar results were also found for a number of breeding selections obtained from crosses between cultivars Picual and Arbequina [33]. However, for some cultivars such as Piñonera, this positive correlation was not so good. Thus, the quite high phenolic content found in Piñonera II fruits (24206.9 µg/g) was not in accordance with the medium-low phenolic content found in the corresponding oil (223.3 µg/g oil). The oils obtained from Picual fruits (stage II) had the highest phenolic content (1267.0 μg/g oil), while the lowest phenolic content was found in oils from cultivar Shengeh (stage IV) with 61.1 μg/g oil.

Pearson’s correlation coefficients were computed using the data obtained from the content of phenolics in the fruits and oils and the data from the *OeAAS* expression levels (Table S2). Significant positive correlation was found between *OeAAS* expression and total fruit phenolics (*r* = 0.63) and even higher correlation coefficients were found for *OeAAS* and total VOO phenolic content (*r* = 0.85). These data are particularly important when taking into account that no clear correlation has been found for other genes/proteins related to the phenolic metabolism in olive. Thus, in a recent study to provide insights about the roles of β-glucosidase, polyphenol oxidase (PPO), and peroxidase (PRX) in the accumulation of phenolic compounds during olive development and ripening [41], it was concluded that *OePPO* and *OePRX* gene expression were not clearly related to the content of secoiridoid compounds. In a similar way, these authors conclude that β-glucosidase gene expression patterns did not parallel either the variations observed in the phenolic content of the corresponding VOOs. It is important to point out that very few genes related to the secoiridoid metabolism in olive have been functionally characterized. In most of the previous studies, putative genes have been selected based on sequence homologies, but further functional evaluation of the encoded proteins is required [42]. In this sense, the data obtained with *OeAAS* seem to be comparable to those reported by Alagna et al. [43] for an olive iridoid synthase gene (*OeISY*), having an expression pattern highly correlated to secoiridoid accumulation that, together with the high specificity of the coded protein for 8-oxogeranial, suggest that OeISY is responsible for the synthesis of the iridoid scaffold in olive.

## 4. Conclusions

Given the undoubted and well-demonstrated functional properties of hydroxytyrosol and the high added value of the hydroxytyrosol derivatives, the identification of the genes and enzymes involved in their synthesis is particularly important both to facilitate new selection tools for olive breeders as to develop synthetic pathways in microbial or plant hosts with metabolic engineering methods. However, very few candidate genes of the secoiridoid biosynthetic pathway have been characterized in olive, and most of them are associated with terpene metabolism [43], but not with the phenylpropanoid metabolism related to the synthesis of the phenolic moiety of the secoiridoid glucosides. By coupling metabolic analyses with transcriptomic data and functional characterization studies, we identified and characterized a highly specific enzyme which catalyzes the conversion of l-DOPA into 3,4-DHPAA, the aldehyde precursor of hydroxytyrosol, in a single step. The catalytic properties of this OeAAS and the expression pattern of its coding gene along the ripening of the fruit in different olive cultivars with divergent phenolic profiles support a key role for OeAAS in the accumulation of hydroxytyrosol-derived secoiridoid compounds in olive fruits. The information provided here could be of great interest for the selection of new olive cultivars with improved secoridoids content through molecular-assisted screening and breeding of olive. In this sense, it is of great interest to search for molecular markers that allow the identification at the seedling stage of genotypes that produce oils with higher levels of secoiridoid compounds.

## Figures and Tables

**Table 1:** Concentration of phenolic compounds, µg/g fresh weight (FW), in olive fruits from Picual, Menya, Shengeh, and Piñonera cultivars harvested at different ripening stages.

PICUAL Phenolics				
(µg/g FW)	Stage I	Stage II	Stage III	Stage IV
Hydroxytyrosol-G	480.6 ± 9.7*	480.7 ± 39.4	283.8 ± 4.0	975.8 ± 30.6
Tyrosol-G	68.5 ± 2.4	68.8 ± 1.8	45.0 ± 0.5	95.5 ± 1.6
Demethyloleuropein	242.7 ± 38.9	82.6 ± 19.5	142.9 ± 0.5	16.4 ± 1.8
Demethyligstroside	159.4 ± 1.2	36.9 ± 16.4	35.6 ± 2.2	22.3 ± 2.6
Oleuropein	30,838.0± 1472.1	27,683.1 ± 1004.0	18,793.2 ± 67.3	7791.4 ± 84.4
Ligstroside	2513.0 ± 242.9	2210.9 ± 144.0	1328.1 ± 19.4	755.0 ± 4.4
Verbascoside	1412.1 ± 253.5	1944.7 ± 173.2	1256 ± 67.6	1816.2 ± 30.6
Luteolin-7-G	677.8 ± 113.5	494.1 ± 69.4	452.1 ± 11.0	180.7 ± 13.9
total phenolics	36,154.3 ± 1014.2	33,735.6 ± 1037.8	22,422.7 ± 122.0	11,738.2 ± 120.1
**MENYA Phenolics**				
**(µg/g FW)**	**Stage I**	**Stage II**	**Stage III**	**Stage IV**
Hydroxytyrosol-G	434.8 ± 27.9	570.4 ± 168.7	463.8 ± 28.9	444 ± 13.7
Tyrosol-G	60.3 ± 6.8	91.1 ± 27.9	52.1 ± 0.8	99.5 ± 0.1
Demethyloleuropein	269 ± 1.6	125.9 ± 20.3	1469.4 ± 385.2	8357.8 ± 416.3
Demethyligstroside	37.9 ± 1.1	40.4 ± 3.1	32.6 ± 6	13.5 ± 6.7
Oleuropein	33,250.5 ± 3011.9	29,839 ± 1634.1	24,269 ± 930.4	2734.2 ± 51.7
Ligstroside	2793.6 ± 341.1	3313.2 ± 76.2	3146.3 ± 247.3	370.7 ± 11.5
Verbascoside	112.1 ± 2.1	123.9 ± 41.9	252.6 ± 44.6	326.6 ± 1.7
Luteolin -7-G	49.9 ± 1.4	146.4 ± 23.1	122.4 ± 28.3	36.6 ± 3.1
total phenolics	37,120.1 ± 988.6	35,247.9 ± 1410.8	30,643.9 ± 1181.9	12,635.4 ± 357.0
**SHENGEH Phenolics**				
**(µg/g FW)**	**Stage I**	**Stage II**	**Stage III**	**Stage IV**
Hydroxytyrosol-G	198.6 ± 15.5	485.5 ± 2.9	685.4 ± 6.4	803.3 ± 92.8
Tyrosol-G	15.1 ± 2.3	28.2 ± 0.5	84.6 ± 0.8	104.3 ± 9.7
Demethyloleuropein	149 ± 7.7	169.7 ± 16.2	25 ± 0.8	209.7 ± 5.9
Demethyligstroside	40.6 ± 0.1	59.6 ± 2.4	44.5 ± 52.7	23.2 ± 0.3
Oleuropein	11,795.3 ± 619.3	6617.5 ± 327.7	3802.7 ± 309.4	4305.1 ± 1
Ligstroside	1227.5 ± 65.1	1005.1 ± 92.1	723.8 ± 44.7	653.3 ± 0.8
Verbascoside	11.7 ± 1.8	98.7 ± 8.5	45.7 ± 16.0	120.5 ± 11.9
Luteolin-7-G	146.4 ± 17.7	163 ± 28.6	106.9 ± 9.3	79.7 ± 8.4
total phenolics	13,512.2 ± 354.4	8866.7 ± 338.6	5738.7 ± 311.2	6364.6 ± 92.5
**PIÑONERA Phenolics**				
**(µg/g FW)**	**Stage I**	**Stage II**	**Stage III**	**Stage IV**
Hydroxytyrosol-G	343.4 ± 78.5	296.6 ± 77	776.1 ± 201.1	690.4 ± 24.7
Tyrosol-G	94.8 ± 49.4	62.2 ± 14.9	104.1 ± 25.7	104.7 ± 0.1
Demethyloleuropein	222.8 ± 24.4	152.9 ± 29.8	1706.2 ± 831.5	8312.8 ± 321.1
Demethyligstroside	224.4 ± 97.4	15.3 ± 0.1	9.5 ± 10.3	3.5 ± 2.9
Oleuropein	25,061.3 ± 2354.7	19,747.3 ± 68.3	15,778.4 ± 1973.9	2537.1 ± 18.7
Ligstroside	3356.9 ± 1208.3	2208.1 ± 10.2	1735.7 ± 399.6	416.9 ± 55.5
Verbascoside	1151.4 ± 95.9	1308.9 ± 405.1	1350.9 ± 72.2	1351.5 ± 52.4
Luteolin-7-G	146.5 ± 34.1	90.1 ± 46.0	169.5 ± 34.3	74.5 ± 10.8
total phenolics	36,488.2 ± 1208.9	24,206.9 ± 460.6	23,404.7 ± 2509.3	13,734.6 ± 343.8

(*) mean contents and standard deviations from three different analyses.

**Table 2:** Concentration of phenolic compounds (µg/g oil) in virgin olive oils (VOOs) obtained from fresh olive fruits (Picual, Menya, Shengeh, and Piñonera cultivars) harvested at different ripening stages.

Cultivar	PICUAL			MENYA			SHENGEH			PIÑONERA		
Phenolics (µg/g)	II	III	IV	II	III	IV	II	III	IV	II	III	IV
Hydroxytyrosol	1.8 ± 0.2*	1.5 ± 0.3	3.2 ± 2.0	1.1 ± 0.2	1.1 ± 0.1	3.4 ± 2.4	0.8 ± 0.1	1.0 ± 0.2	1.5 ± 0.0	0.5 ± 0.5	1.0 ± 0.1	1.6 ± 0.0
Tyrosol	3.4 ± 1.0	1.9 ± 0.1	3.6 ± 0.7	4.1 ± 0.2	3.8 ± 0.1	6.2 ± 0.8	2.50 ± 0.5	3.4 ± 0.7	4.1 ± 1.0	3.8 ± 0.0	3.4 ± 0.1	5.5 ± 0.2
Vanillic acid	0.5 ± 0.1	0.2 ± 0.0	0.4 ± 0.4	0.5 ± 0.0	0.4 ± 0.0	0.6 ± 0.0	0.6 ± 0.1	0.7 ± 0.2	0.6 ± 0.1	1.5 ± 0.0	0.1 ± 0.0	0.1 ± 0.0
Vainillin	0.2 ± 0.0	0.1 ± 0.0	0.1 ± 0.1	0.3 ± 0.2	0.3 ± 0.0	0.2 ± 0.0	0.2 ± 0.0	0.3 ± 0.1	0.2 ± 0.0	0.2 ± 0.0	0.1 ± 0.0	0.3 ± 0.0
*p*-Coumaric acid	1.2 ± 0.1	0.9 ± 0.1	0.4 ± 0.1	0.2 ± 0.0	0.2 ± 0.0	0.1 ± 0.0	0.6 ± 0.1	0.4 ± 0.1	0.4 ± 0.1	0.6 ± 0.0	0.5 ± 0.1	0.4 ± 0.0
Hydroxytyrosol ac.	0.8 ± 0.1	0.3 ± 0.0	1.6 ± 0.3	1.3 ± 0.1	2.4 ± 0.6	7.5 ± 0.1	1.3 ± 0.1	2.4 ± 0.8	0.3 ± 0.3	2.6 ± 0.1	3.8 ± 0.0	7.8 ± 0.3
3.4-DHPEA-EDA	40.9 ± 3.4	39.2 ± 1.1	19.5 ± 1.4	83.69 ± 1.6	121.3 ± 13.4	256.6 ± 4.7	15.6 ± 0.9	15.2 ± 1.1	6.4 ± 1.5	68.9 ± 1.6	59.6 ± 4.4	98.0 ± 3.6
p-HPEA-EDA	46.3 ± 0.1	36.9 ± 4.6	16.7 ± 2.9	90.0 ± 10.1	87.6 ± 2.7	171.5 ± 2.4	20.7 ± 1.3	20.6 ± 2.7	5.1 ± 1.2	102.7 ± 6.7	60.7 ± 2.0	69.4 ± 6.3
Pinoresinol	12.7 ± 0.5	9.3 ± 0.7	3.4 ± 0.7	7.7 ± 0.2	6.2 ± 0.2	3.6 ± 0.1	2.8 ± 0.3	2.9 ± 0.2	1.7 ± 0.4	2.5 ± 0.1	2.1 ± 0.0	2.4 ± 0.1
Cinnamic acid	1.7 ± 0.2	1.3 ± 0.3	0.3 ± 0.1	2.2 ± 0.6	2.2 ± 0.2	0.9 ± 0.0	0.2 ± 0.0	0.3 ± 0.0	0.1 ± 0.0	0.4 ± 0.0	0.4 ± 0.0	0.6 ± 0.0
Acetoxypinoresinol	37.1 ± 0.3	32.1 ± 0.7	14.6 ± 5.9	61.4 ± 05	47.7 ± 0.5	22.1 ± 2.1	9.8 ± 0.7	9.6 ± 0.7	6.8 ± 1.5	4.9 ± 0.0	4.4 ± 0.0	4.5 ± 0.1
3.4-DHPEA-EA	1068.6 ± 15.7	854.7 ± 26.4	179.7 ± 5.5	425.0 ± 41.3	388.8 ± 7.3	162.9 ± 5.3	19.2 ± 3.0	37.9 ± 3.4	21.6 ± 2.3	22.1 ± 1.3	11.9 ± 2.0	14.5 ± 0.8
*p*-HPEA-EA	42.3 ± 1.6	37.1 ± 6.6	16.8 ± 2.0	65.1 ± 3.5	40.0 ± 4.3	19.2 ± 0.9	7.5 ± 0.5	9.0 ± 1.1	6.6 ± 1.1	10.4 ± 0.6	5.9 ± 0.0	5.6 ± 0.0
Ferulic acid	0.1 ± 0.0	0.0	0.2 ± 0.1	0.0	0.0	0.1 ± 0.0	0.1 ± 0.0	0.1 ± 0.0	0.1 ± 0.0	0.1 ± 0.0	0.1 ± 0.0	0.1 ± 0.0
Luteolin	8.1 ± 1.8	8.3 ± 0.1	5.8 ± 1.7	1.9 ± 0.6	1.5 ± 0.0	4.4 ± 0.3	3.7 ± 0.7	4.1 ± 0.2	5.0 ± 0.2	1.7 ± 0.1	2.3 ± 0.0	1.9 ± 0.0
Apigenin	1.9 ± 0.4	1.9 ± 0.1	1.1 ± 0.4	0.4 ± 0.1	0.2 ± 0.0	0.6 ± 0.0	0.7 ± 0.2	0.6 ± 0.1	0.6 ± 0.0	0.2 ± 0.0	0.3 ± 0.0	0.2 ± 0.0
total phenolics	1267.7 ± 20.0	1025.8 ± 20.9	267.5 ± 3.0	744.8 ± 69.0	703.8 ± 19.6	659.8 ± 0.7	86.4 ± 2.1	108.6 ± 4.8	61.1 ± 8.8	223.3 ± 10.8	156.6 ± 4.4	212.9 ± 11.2
total *o*-phe	1120.3 ± 17.5	904.1 ± 27.9	209.9 ± 4.4	513.0 ± 55.7	515.1 ± 8.8	434.8 ± 3.4	40.6 ± 3.1	60.5 ± 1.0	34.8 ± 3.3	95.8 ± 3.4	78.6 ± 2.4	123.8 ± 4.6
secoiridoids	1198.2 ± 20.6	967.9 ± 25.8	232.8 ± 3.0	663.8 ± 68.3	637.7 ± 15.4	610.3 ± 0.9	63.0 ± 4.6	82.7 ± 1.5	39.8 ± 6.0	204.1 ± 10.1	138.1 ± 4.6	187.5 ± 10.5

(*) mean contents and standard deviations from three different analyses.

## Supplementary Materials

The following are available online https://www.mdpi.com/2076-3921/8/9/352/s1. Figure S1: Multiple amino acid sequence alignment of the four olive AAAD genes identified by a transcriptomic analysis and the *R. rosea* and *P. crispum* AAS genes; Figure S2: Phylogenetic tree illustrating relatedness of *OeAAS* to other plant AADCs identified as TyDC and AAS; Table S1: Real Time-Quantitative PCR primers used in this work; Table S2: Pearson’s correlation coefficients among *Oe*AAS expression levels and the main phenolic compounds found in fruits and the total phenolic content in VOO.
