# Supplementary material for: An Aromatic Aldehyde Synthase Controls the Synthesis of Hydroxytyrosol Derivatives Present in Virgin Olive Oil
Source: Antioxidants (Basel). 2019 Sep 1;8(9):352. doi: 10.3390/antiox8090352 (PMC6770214; doi:10.3390/antiox8090352)
Supplement: Supplementary file 1 [file antioxidants-08-00352-s001.pdf]

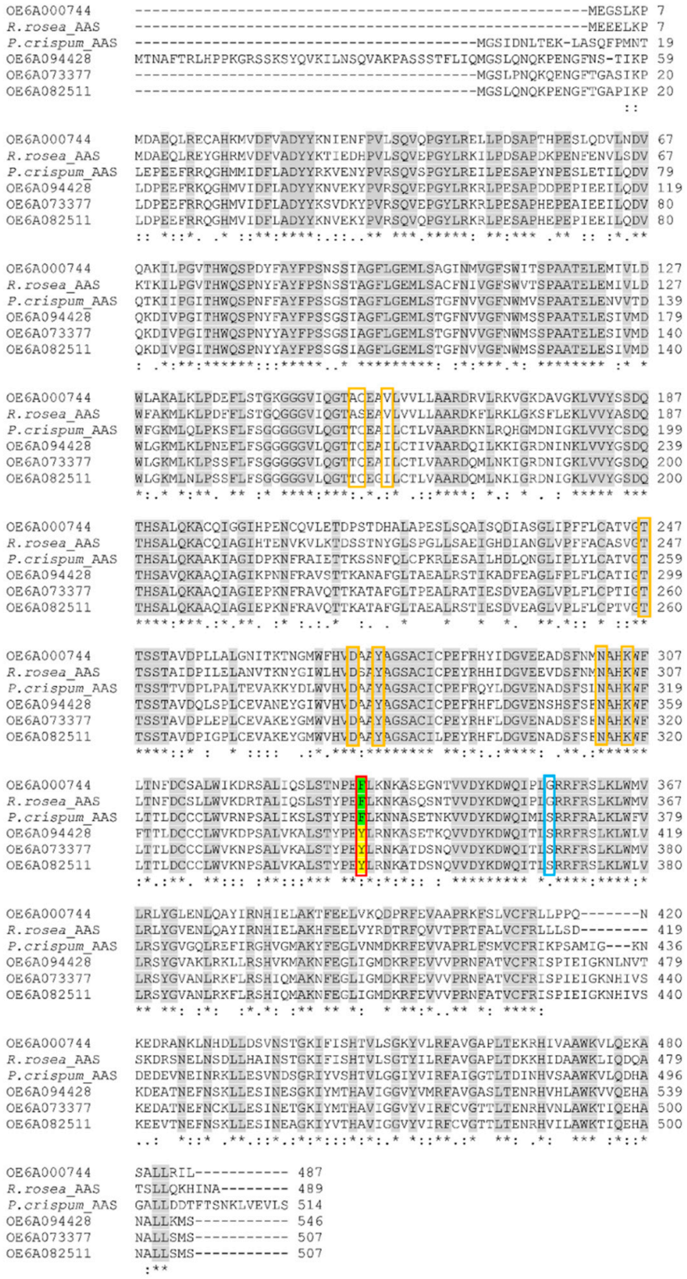

Figure S1. Multiple amino acid sequence alignment of the four olive AAAD genes identified by a transcriptomic analysis and the *R. rosea* and *P. crispum* AAS genes. The highly conserved residues are marked with and asterisk and shaded. Aminoacids involved in pyridoxal 5'-phosphate binding site [36] are shown in orange squares. Indolic/Phenolic substrate-specificity dictating aminoacid [24] is squared in blue. Activity-dictating residue [23] is squared in red: highlighted in green the phenylalanine for the aldehyde synthase activity and highlighted in yellow the tyrosine for the decarboxylation activity.

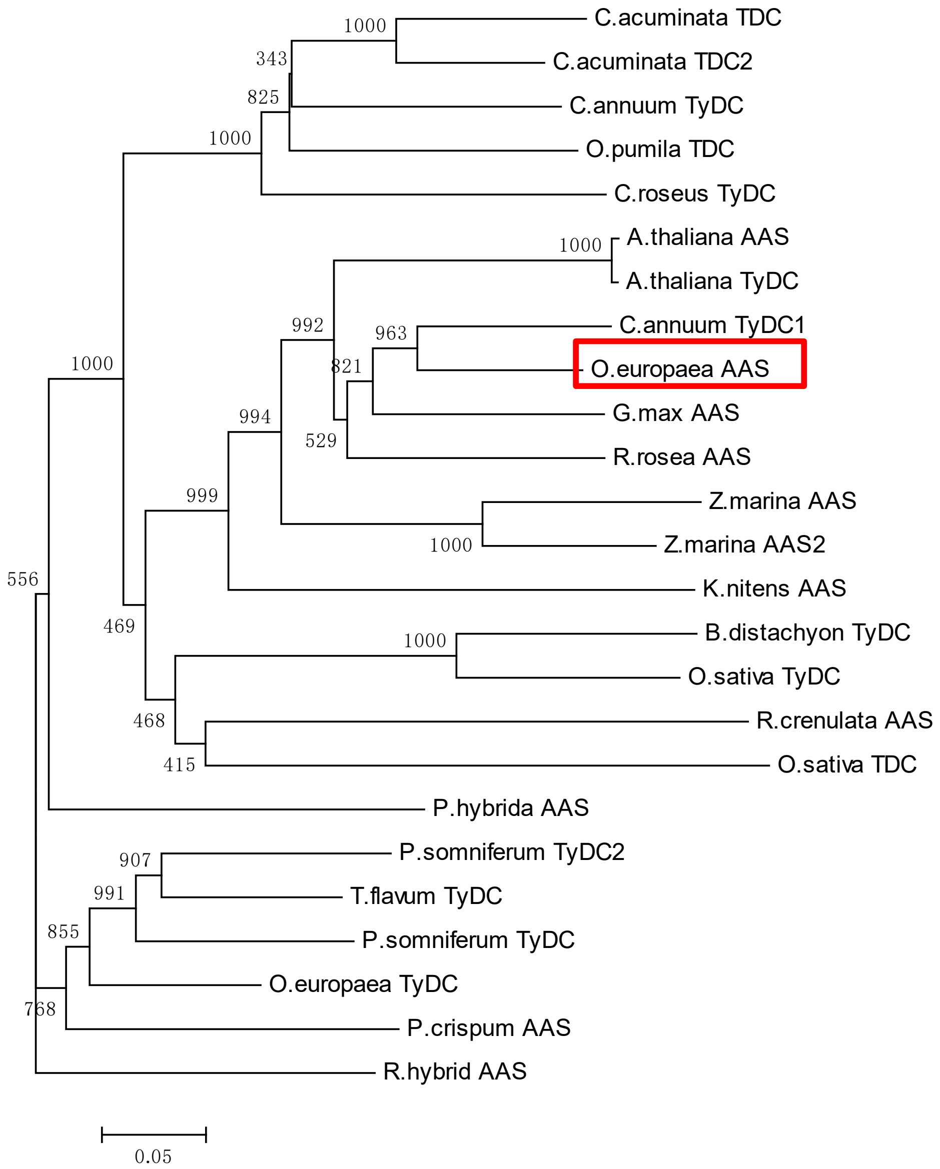

Figure S2. Phylogenetic tree illustrating relatedness of *OeAAS* to other plant AADCs identified as TyDC and AAS. Accession numbers of the different AAS/TyDC included in the analysis: A.thaliana_AAS ADV41492.1; A.thaliana_TyDC CAB56038.1; B.distachyon_TyDC XP_003569907.1; C.acuminata_TDC AAB39708.1; C.acuminata_TDC2 AAB39709.1; C.annuum_TyDC NP_001312016.1; C.roseus_TyDC sp|P17770.1; C.annuum_TyDC XP_016541857.1; G.max_AAS XP_006576967.1; K.nitens_AAS GAQ86385.1; Olea europaea TyDC AFS28699.; O.pumila_TDC BAC41515.1; O.sativa_TyDC XP_015633932.1; O.sativa_TDC XP_015648701.1; P.crispum_AAS sp|Q06086.1; P.hybrida_AAS ABB72475.1; P.somniferum_TyDC AAC61842.1; P.somniferum_TyDC2 sp|P54769.1|; R.crenulata_AAS AFN89854.1 tyrosine decarboxylase [Rhodiola crenulata]; R.hybrid_AAS ABB04522.1 phenylacetaldehyde synthase [Rosa hybrid cultivar]; R.rosea_AAS AUI41112.1; T.flavum_TyDC AAG60665.1; Z.marina_AAS KMZ74011.1; Z.marina_AAS2 KMZ74017.1.

Table S1. Real Time-Quantitative PCR primers used in this work.

Primer	Sequence (5′→ 3′)
qOeAAS-F	GTTGCAGCTTGGAAGGTGTT
qOeAAS-R	GGGCCTGCTCCTATGTATCA
qOeEF1α-F	TGCTCTATCTGGATTGCCATT
qOeEF1α-R	TCAAATGCCACCATGACTTC
qOeGAPDH-F	TGAGATGCTGCACAATGGTT
qOeGAPDH-R	CACGATAGGCTTACGCAACA
qOePP2A-F	CTCGCCTGAAAACGAAAGAC
qOePP2A-R	CACAAAGCAGACCAAAACCA

Table S2. Pearson's correlation coefficients among *Oe*AAS expression levels and the main phenolic compounds found in fruits and total phenolic contents in VOO.

	**Hty-G**	**Tyr-G**	**DemO**	**DemL**	**Oleuropein**	**Ligstroside**	**Verbascoside**	**Lut-7-G**	**Total Phe FRUIT**	**Total Secoir. FRUIT**	***OeAAS***	**Total Phe VOO**	**Total Secoir VOO**
Hty-G	1.000000	0.716646	-0.025626	-0.146194	-0.319033	-0.244317	0.058980	-0.292579	-0.355155	-0.353229	-0.347160	-0.496267	-0.501708
Tyr-G	0.716646	1.000000	0.358126	-0.455802	-0.205318	-0.174080	0.073007	-0.398602	-0.123907	-0.114411	-0.410502	-0.263025	-0.266471
DemO	-0.025626	0.358126	1.000000	-0.513434	-0.449691	-0.406724	-0.013583	-0.422268	-0.195159	-0.185757	-0.329693	-0.042156	-0.039392
DemL	-0.146194	-0.455802	-0.513434	1.000000	0.122066	0.136563	-0.393577	0.276111	-0.055534	-0.019661	0.331025	0.094682	0.087882
Oleuropein	-0.319033	-0.205318	-0.449691	0.122066	1.000000	0.936195	0.234250	0.544884	0.960140	0.960585	0.654101	0.676250	0.669078
Ligstroside	-0.244317	-0.174080	-0.406724	0.136563	0.936195	1.000000	0.010662	0.263481	0.890882	0.916306	0.473319	0.462645	0.450116
Verbascoside	0.058980	0.073007	-0.013583	-0.393577	0.234250	0.010662	1.000000	0.547687	0.310044	0.231174	0.170202	0.295214	0.312247
Lut-7-G	-0.292579	-0.398602	-0.422268	0.276111	0.544884	0.263481	0.547687	1.000000	0.487450	0.444162	0.720279	0.735444	0.745594
Total Fruit- phe	-0.355155	-0.123907	-0.195159	-0.055534	0.960140	0.890882	0.310044	0.487450	1.000000	0.993997	0.631042	0.735599	0.729419
Total Fruit seco	-0.353229	-0.114411	-0.185757	-0.019661	0.960585	0.916306	0.231174	0.444162	0.993997	1.000000	0.603744	0.712955	0.705320
OeAAS	-0.347160	-0.410502	-0.329693	0.331025	0.654101	0.473319	0.170202	0.720279	0.631042	0.603744	1.000000	0.851364	0.844856
Total Phe VOO	-0.496267	-0.263025	-0.042156	0.094682	0.676250	0.462645	0.295214	0.735444	0.735599	0.712955	0.851364	1.000000	0.999572
Total Sec VOO	-0.501708	-0.266471	-0.039392	0.087882	0.669078	0.450116	0.312247	0.745594	0.729419	0.705320	0.844856	0.999572	1.000000

Correlations marked in red are significant at $p < 0.05$ ($n = 36$).

© 2019 by the authors. Licensee MDPI, Basel, Switzerland. This article is an open access article distributed under the terms and conditions of the Creative Commons Attribution (CC BY) license (http://creativecommons.org/licenses/by/4.0/).
